# The Efficacy and Safety of Topiramate for Prophylaxis of Migraine in Children

**Published:** 2013

**Authors:** Razieh FALLAH, Sedighah AKHAVAN KARBASI, Ahmad SHAJARI, Mostafa FROMANDI

**Affiliations:** 1 Growth Disorders of Children Research Center, Shahid Sadoughi University of Medical Sciences, Yazd, Iran; 2 Department of Pediatrics, Shahid Sadoughi University of Medical Sciences, Yazd, Iran; 3 Department of Pediatrics, Aliebn-Abitaleb School of Medicine, Islamic Azad University, Yazd Branch, Yazd, Iran

**Keywords:** Headache, Migraine, Prophylaxis, Topiramate

## Abstract

**Objective:**

Migraine is the most common acute intermittent primary headache in children and prophylactic therapy is indicated in children with frequent or disabling headaches. The purpose of this study was to evaluate the efficacy and safety of topiramate (TPM) for migraine prophylaxis in children.

**Materials & Methods:**

In a quasi-experimental study, monthly frequency, severity and duration of headache, migraine disability, and side-effects were evaluated in 100 children who were referred to the Pediatric Neurology Clinic of Shahid Sadoughi University of Medical Sciences, Yazd, Iran from April 2011 to March 2012, and were treated with 3 mg/kg/day of TPM for three months.

**Results:**

Fifty eight (57.4%) girls and 42 (41.6%) boys with the mean age of 10.46±2.11 years were evaluated. Monthly frequency, severity, and duration of headache decreased with treatment from 15.34±7.28 to 6.07±3.16 attacks, from 6.21±1.74 to 3.15±2.22, and from 2.28±1.55 to 0.94±0.35 hours, respectively, and the Pediatric Migraine Disability Assessment score reduced with TPM from 32.48±9.33 to 15.54±6.16. Transient side-effects were seen in 21% of the patients, including hyperthermia in 11%, anorexia and weight loss in 6%, and drowsiness in 4%. No serious side-effects were reported.

**Conclusion:**

TPM could be considered as a safe and effective drug in pediatric migraine prophylaxis.

## Introduction

Migraine is the most common acute intermittent primary headache in children that occurs in up to 10.6% of 5-15-year-old children (1).

During the past fifty years, several diagnostic criteria for pediatric migraine have been proposed and currently, second edition of the International Classification of Headache Disorders (ICHD-II) for children migraine that was published by the International Headache Society in 2004 has been accepted (2).

Preventive or prophylactic therapy is used in children with frequent (one or more headaches per week) or disabling (missing school, home or social activities, or a Pediatric Migraine Disability Assessment score (pedMIDAS) above 20) headaches (1).

Preventive medications, including calcium channel blockers, cyproheptadine, beta blockers and anticonvulsants have been used in children (3).

Recently, antiepileptic drugs are more commonly used in adults and adolescents for migraine prophylactic therapy (1).

Topiramate (TPM) is an antiepileptic drug that the exact mechanism of its effectiveness in migraine is unclear. However, its migraine preventive effect may be based on the following mechanisms:

a) inhibition of excitatory neurotransmission by blocking glutamate at a-amino-3-hydroxy-5-methylisoxazole-4-propionic acid–kainate receptors b) enhancing the inhibitory activity of γ-aminobutyric acid receptors

c) blocking voltage-dependent sodium channels

d) blocking high-voltage–activated calcium channels

e) inhibition of erythrocyte carbonic anhydrase (4).

Safety of TPM in Iranian epileptic children has been reported as well (5). There are limited studies about efficacy of TPM for migraine prevention in children in Iran.

The purpose of this study was to evaluate the efficacy and safety of TPM for migraine prophylaxis in children in Yazd, central city of I.R. Iran.

## Materials & Methods

This quasi-experimental (before and after) study was conducted on 5-15-year old migraineurs, who were referred to Pediatric Neurology Clinic of Shahid Sadoughi University of Medical Sciences, Yazd, Iran from April 2011 to June 2012. 

Sample size was assessed to be 100 children based on Z formula and a confidence interval of 95% with 80% power to detect a 20% difference in efficacy between the two groups with type one error (alpha) of 0.05. 

Eligible participants included those children aged 5-15 years, had migraine headache based on ICHD-II criteria (2) in clinical evaluation by pediatric neurologist for at least six months before the study, used no migraine preventive therapy, and had frequent (one or more headache attacks per week) or disabling (PedMIDAS more than 20) headaches that prophylactic therapy was indicated for them.

Exclusion criteria consisted of presence of metabolic acidosis, kidney dysfunction, renal stone, any serious systemic diseases, headaches other than migraine and secondary headaches, or those who did not complete the three-month period of treatment.

Headaches other than migraine, secondary headaches, and systemic diseases were excluded by pediatric neurologist clinical evaluation of patients through taking history, physical examination, laboratory evaluation if indicated, and brain magnetic resonance imaging when increased intracranial pressure was suspected, either by historical suspicion or physical exam (1).

Patients were treated with 3 mg/kg/day of TPM in two divided doses. Children were visited for three consecutive months and clinical information about frequency, severity, and duration of headaches, pedMIDAS score (6) and drug side-effects were recorded.

Severity of headache was assessed by asking each child to grade majority of headache pain on visual analogue scale (VAS) (7) on a 10-point scale as no pain=scale of 0 and the most severe pain=10. A VAS is a horizontal or vertical 10-cm length line that is marked at the extremes by “no pain” and “worst pain imaginable”. The children were asked to place a mark on the line that showed their pain level.

Monthly frequency, severity and duration of headache, and pedMIDAS were evaluated before and three months after TPM therapy.

More than 50% of reduction in monthly headache frequency was considered as good response. Clinical side-effects of TPM in the duration of treatment were recorded through interviewing the parents of the patients. 

The data were analyzed using SPSS 17 statistical software. Chi-square test was used for analysis of qualitative variables and mean values were compared using T-test. Differences were considered significant at p-values of less than 0.05.

Informed consent was taken from patients’ parents and the study has been approved by the Ethics Committee of Shahid Sadoughi University of Medical Sciences, Yazd, Iran.

The researchers got no support from drug companies. 

## Results

Fifty eight (57.4%) girls and 42(41.6%) boys with the mean age of 10.46±2.11 years were evaluated. Onset age of headache was 4-14 years (mean±SD: 9.96±8.16 years). After three months of treatment, good response (more than 50% reduction in monthly headache frequency) was seen in 74% of children (95% confidence interval of 0.71-0.93). 

Frequency distribution of the good response based on some characteristics of the children is shown in Table 1, which indicates that no statistically significant differences were seen in terms of sex distribution, migraine type, positive family history of migraine, mean of age, and mean of onset age of migraine between the two groups.

Table 2 shows headache characteristics before and after TPM treatment which indicates that the drug was effective in reduction of monthly frequency, severity, duration, and disability of headache. 

No serious adverse events were seen in the patients, but transient and mild clinical side-effects were observed in 21% (N=21) of children, including hyperthermia in 11, anorexia and weight loss in 6, and drowsiness in 4 children. All side-effects disappeared in one or two weeks and treatment was stopped in none of the patients who suffered from the side-effects.

## Discussion

Various drugs have been used for migraine prophylaxis in children. In the present study, efficacy and safety of TPM was evaluated and the results showed that TPM is effective in the reduction of monthly frequency, severity, duration, and disability of headache which is in agreement with another Iranian study on adult migraineurs. In this study, duration of headache decreased from 2.28±1.55 to 0.94±0.35 hours, but in a study by Ashtari et al. in Isfahan, Iran, headache duration decreased with TPM treatment from 16.37±7.26 to 6.23±5.22 hours (9). In children, duration of migraine headache was shorter than adult (2).

In the present study, more than 50% of the reduction in monthly headache frequency was attained with TPM in 74% of patients. However, in other studies, the proportion whose monthly headache frequency decreased more than 50% varied from 55% to 100% (10-15). Possible explanations for these discrepancies are differences in age, drug dosage, race, sample size, and design of study. 

In this study, TPM was effective in improvement of headache disability by reduction of pedMIDAS score which is in agreement with other studies (16,17). 

In our study, both drugs were safe and no serious clinical adverse event was seen in the two groups. Safety of TPM has also been reported in other researches (10,11,17,18).

In this study, in children taking TPM, side effects including hyperthermia in 11%, anorexia and weight loss in 6% and drowsiness in 4% were seen and all of these side effects were mild and transient and not significant enough to exclude from the study. Weight loss was the most common side-effects in two other studies (10,17).

In Lewis et al.’s study, upper respiratory tract infection, paresthesia, and dizziness were more frequently in TPM than placebo group (11).

In a study in Barcelona, 33.3% of children who were treated with TPM showed side-effects, none of which were serious (13).

In Winner et al.’s study, upper respiratory tract infection, anorexia, weight loss, gastroenteritis, paresthesia and drowsiness were more frequently in TPM than placebo group (10).

In Cruz et al.’s study, 27% of 7.3-20.5 year migraineurs who were treated with TPM had side-effects and drug dose was more in patients with adverse effects (2.8±1.5 mg/kg/day vs. 1.27±0.7 mg/kg/day) (14). 

In an Indian study, weight loss, reduced concentration in school, drowsiness, and parasthesia were more important adverse effects of TPM (17).

TPM may also cause cognitive and concentration dysfunction (17, 19).

The limitations of the present study were lack of placebo and no assessment of children’s cognitive function. 

**Table 1.** Frequency Distribution of The Good Response Based on Some Characteristics of Children

Good response		Yes (N=74)	No (N=26)	p-value
Data				
Sex	Girl	43	15	0.97
	Boy	31	11	
Type of migraine	Without aura	46	15	0.44
	With aura	25	11	
Positive family history of migraine	Yes	69	20	0.14
	No	5	6	
Age in year (mean ±SD)		10.30±2.17	10.92±2.24	0.22
Onset age of migraine (mean ±SD)		8.25±2.36	8.92±2.01	0.20

**Table 2:** Headache Characteristics Before and After Topiramate Treatment

Time	Before treatment (Mean ± SD)	After treatment (Mean ± SD)	p-value
Data			
Monthly headache frequency	15.34±7.28	6.07±3.16	0.0001
Severity of headache	6.21±1.74	3.15±2.22	0.001
Headache duration in hours	2.28±1.55	0.94±0.35	0.002
Headache disability: pedMIDAS	32.48±9.33	15.54±6.16	0.0001

## Notes

**In conclusion**, based on results of the present study, TPM is a safe and effective drug for pediatric migraine prophylaxis in reduction of monthly frequency, severity, duration, and disability of migraine headache in children.
